# Measuring global functioning in older adults with cognitive impairments using the Rasch model

**DOI:** 10.1186/s12877-020-01886-0

**Published:** 2020-11-23

**Authors:** Rocco Palumbo, Alberto Di Domenico, Federica Piras, Salvatore Bazzano, Mario Zerilli, Fabio Lorico, Erika Borella

**Affiliations:** 1 Department of Psychological, Health and Territorial Sciences, University of Chieti, Via dei Vestini 31, 66100 Chieti, Italy; 2 Department of Neurology, Boston University, School of Medicine, Boston, MA USA; 3 IRCCS Santa Lucia Foundation, Neuropsychiatry Laboratory, Clinical and Behavioral Neurology Department, Rome, Italy; 4 Centro Decadimento Cognitivo Asl7 di Bassano del Grappa, Bassano del Grappa, Italy; 5 Department of General Psychology, University of Padova, Padova, Italy

**Keywords:** Rasch model, Cognitive impairment, MCI, Global functioning, Activities of daily living

## Abstract

**Background:**

Cognitive and functional measures are often measured and interpreted separately during the clinical evaluation of patients with cognitive impairment. This can sometimes lead to a challenging interpretation when measures do not show concordance, especially after a clinical intervention. In this study, the development and evaluation of a new approach, using the Rasch model, that combines cognitive and functional measures in one single and more powerful measure (compared to stand-alone tests) to assess global functioning in older adults with cognitive impairment (including dementia) was presented.

**Methods:**

Clinical data from 265 older adults’ subjects diagnosed with mild cognitive impairment, or dementia, included: The Mini-mental state examination (MMSE), the Esame Neuropsicologico Breve (ENB) – a neuropsychological battery used in Italy–, the Activities of Daily Living (ADL), and the Instrumental Activities of Daily Living (IADL) questionnaires.

**Results:**

Patients with severe cognitive impairment showed lower global functioning score compared to patients with moderate impairment. Receiver Operating Characteristic (ROC) curve analyses were performed to determine sensitivity and specificity of the global functioning score resulting from the combined measure. Results showed that the global functioning score discriminates better between patients with severe and moderate cognitive impairment compared to the ENB, ADL, and IADL when considered separately.

**Conclusions:**

The Rasch model was able to combine cognitive and functional measures into a single score (global functioning score). All together, these results suggest that the diverse cognitive and functional measures can be considered part of one single dimension (global functioning) and that this dimension can be measured as a single construct and score. This study offers an alternative perspective for future development of instruments that would help clinicians in measuring global functioning in older adults’ patients at different stages of cognitive impairments and different baseline level of performance.

## Background

Assessing the severity of cognitive impairment, or the outcome of a cognitive intervention, is a priority when developing patient-oriented strategies for functional amelioration [1, 2].

The current gold standard for detection and staging-oriented assessment of cognitive impairment in older adults comprises neuropsychological tests sensitive to early cognitive impairments, performance-based assessment that provides standardized and objective means to assess deficits in functional capacity, and related imaging biomarkers [3–5]. Outcome assessments, especially in elderly people with MCI or in the pre-MCI phase, should be able to detect both, stability and worsening of cognitive and functional abilities in subjects that have considerable variations in their baseline level of performance. Sensitivity to long-term stability is a particularly critical goal for measurements in prevention interventions, as these may not improve cognitive or functional performance, but may prevent progression to overt dementia. Concurrently, the capacity to detect developing impairments in cognition and functioning is crucial in preclinical and early dementia stages samples, as both, the level of difficulty of the assessment and its sensitivity to decline, are critical aspects to capture subtle markers of incipient deterioration [6–8].

The actual and mainly used assessment approach provides multiple outcome measures that can lead to contradictory outcomes, thus confusing the evaluation of the interventions’ benefits [9]. The most common method to measure and monitor disease severity consists in calculating a total score that is often derived from the sum of all items of the single test that is administered (e.g. Mini Mental State Examination). Although this seems to be a simple method, it could lead to an inaccurate estimation of cognitive functioning [10], because it assumes that all the items of a cognitive/functional test have the same difficulty and are interchangeable [11]. The level of difficulty, even at the single item level, depends and varies as a function of diagnosis, since a particular skill might be completely intact in pre-MCI, impaired in MCI, and fully deteriorated in AD. Therefore, the total raw score of a test often does not reflect the real cognitive status and profile of an individual. Moreover, if a treatment or intervention is administered, potential benefits might be masked as the gains are based on the comparison between the pre and post-intervention total raw score of a given test. Yet, the patient could have improved on certain domains measured by the test, and declined in others.

The increasing number of studies that try to develop a tailored item banking using Rasch analysis supports the need for a more sensitive assessment tool in older adults with cognitive impairment [12–15].

Rasch analysis is part of Item Response Theory that is a modern psychometric test theory assuming the existence of a single common factor - the latent trait, which accounts for the covariance of all items. The Rasch model has one parameter for the person (ability), and one parameter corresponding to each category of an item (difficulty). Rasch analysis involves a simple data transformation, which determines where items and respondents are located on a linear scale. Additionally, it provides a hierarchical order of each item based on level of difficulty, as well as an ability score for each subject. The latter is based on each subject’s performance: the more difficult the item that the subject performs correctly, the higher the ability score, and vice versa [16–19]. Although Rasch analysis has been widely applied to create improved instruments from well-validated questionnaires [20–26], and despite the increasing number of studies trying to develop a composite score (using both cognitive and functional measures) [27–30], no study, at least to our knowledge, has used the Rasch model to develop a comprehensive instrument derived from the combination of different items of different tests for measuring global functioning.

In this study we propose the development and evaluation of a new approach, using the Rasch model, for measuring global functioning (that is the overall ability of patients to function in their everyday activities) in older adults with cognitive impairment and dementia. The main aim is to obtain a global functioning score that can have the potential to provide a single score, based on subjects’ performance and the items’ level of difficulty, representative of the individual’s global functioning and that can define the range of cognitive impairment severity levels. Indeed, a key characteristic of endpoints, used in clinical studies, is that they should closely and comprehensively denote the overall disease by being both, sensitive and discriminative among different levels of impairment. This single score will be easily comparable at different time points and across the range of different impairment levels. In order to test our hypothesis, different types of measures (cognitive and functional) were combined using the Rasch model. The advantage of using the Rasch model to create a composite score consists in combining cognitive and functional measures taking into account, at the same time, the level of difficulty of each item in the considered tests.

The rationale behind this approach assumes that different cognitive abilities, required to perform both, cognitive tasks and daily activities (e.g. memory, verbal fluency, attention, getting dressed, shopping, doing laundry, etc.) [31–33], are all part of one single dimension (global functioning) which can be represented on one single linear scale. It is, therefore, expected that the probability of performing well on a task will increase monotonically with the difference between that person’s cognitive ability and the level of cognitive ability required for that task.

If this hypothesis is verified, this study will provide the possibility of developing a comprehensive assessment tool able to discriminate the patient’s cognitive/functional ability, producing an outcome scale that, starting from different type of measures, will provide a single score (global functioning) also sensitive to potential changes after treatment.

Data from a standardized battery of cognitive and functional tests, administered to 265 older adults with a diagnosis of MCI or dementia, were submitted to the Rasch model to derive a single score potentially able to discriminate patients’ cognitive ability. Concurrently, by testing the possibility of combining cognitive and functional tests in a single outcome score, we tested whether the resulting composite score is a better predictor of cognitive impairment, compared to stand-alone tests. In order to test our hypothesis, different types of measures (cognitive and functional) were combined using the Rasch model.

## Methods

### Participants

This study is based on a retrospective analysis on clinical data from patients that received clinical care at the ASL of Bassano, Italy, between 2013 and 2018. Only patients with clinical diagnosis of MCI, or dementia, and without functional impairment due to physical inability, were included in this study. We excluded individuals that did not complete the neuropsychological assessment which comprises a very common battery of tests used in Italy: the *Esame Neuropsicologico Breve* (ENB, Short Neuropsychological Examination [34];, as well as the Activity of Daily Living (ADL [35];, the Instrumental Activities of Daily Living (IADL [36]; questionnaires, and the Mini-Mental State Examination (MMSE [37, 38];.

A total of 265 medical records were included in the following analyses. All individuals were outpatients in the age range of 62 to 95 (M = 78.25, SD = 5.80) with education between 0 and 18 years (M = 7.09, SD = 3.60). All patients were able to read and write at the time of the testing.

### Measures

The MMSE comprises items that test temporal and spatial orientation, immediate and delayed verbal memory, language, attention, and praxis. The dependent variable was the sum of the items’ scores (max. 30), corrected for age and education.

*Esame Neuropsicologico Breve* (ENB, Short Neuropsychological Examination [34];).

The ENB encompasses 16 tasks. The following description of each test reflects the order of the administration.

- 1) *Digit span*: it consists of seven pairs of random number sequences of digits (from 2 to 8). The examiner reads each sequence, one at a time, and the participant has to repeat it in the same order (forward digit span). The test continues until the participant is unable to repeat the sequence correctly. The test assesses short-term memory storage capacity to passively retain verbal information for a few seconds. Only sequences containing between 3 and 8 digits were administered in this study.

- 2) *Trail Making Test A (TMT A) and 3) Trail Making Test B (TMT B)*: TMT *A* consists of encircled numbers from 1 to 25 randomly distributed on a sheet of paper. The task consists in connecting the numbers from 1 to 25 as quickly as possible. This test assesses psychomotor speed and attention. *TMT B* is more complex because it requires the subject to connect numbers and letters in an alternating pattern (1-A-2-B-3-C, etc.). TMT B evaluates attention, switching ability, and working memory (i.e. executive functions).

- 4) *Copy drawing*: the subject is asked to copy a picture. This test evaluates constructional abilities.

- 5) *Interference memory (10 s., A) and 6) Interference memory (30 s., B)*: the test is based on the Brown-Peterson paradigm with the aim to prevent rehearsal of material held for short-term retention. Upon seeing a trigram of consonants, the subject is required to count forward by two from a given number until instructed to stop, and then report the trigram. For the first three items the subject has to count for 10 s (A), and for the following three items for 30 s (B). While in the first case (A), mainly executive functions are involved (i.e. distribution of attention on two simultaneous tasks), in the second case (B) memory is also required.

- 7) *Abstract verbal reasoning:* three pairs of words with different degrees of abstraction are presented to the subject who is required to find out the common superordinate category (e.g., *What do pasta and milk have in common?* The expected answer is *they are types of food*). The test assesses subjects’ ability to find commonalities among different concepts on an abstract level. Abstract thinking is part of executive functions.

- 8) *Token test:* a set of 10 plastic tokens of five different colors and two different shapes (circles and squares) are placed in front of the subject. Five verbal commands are given to the subject (e.g., *Please touch the red circle and the green circle*) to assess his/her verbal comprehension.

- 9) *Immediate Story recall test* (a): the examiner reads the story and then asks the subject for immediate recall;

- 10) *Delayed Story recall* (b): the examiner reads the story a second time and tests memory again after a five-minute delay. During this five-minute interval another test (*Overlapping Figures*) is administered. In both versions (a) and (b) the recall is free, thus the order of reporting the different information units is not relevant, but the logical meaning and the relations between units have to be maintained. This test evaluates essential memory functions.

- 11) *Overlapping Figures*: the participant is shown a black and white visual pattern of many overlapping figures. Numbers, letters, animals, human figures, and objects of different dimensions can be visually recognized. The subject is required to indicate and name as many figures as possible within a four-minute period. The test verifies the ability to recognize shapes from a background (i.e. cognitive flexibility in selecting overlapping shapes inhibiting the ones already recognized). Thus, it requires the involvement of executive functions.

- 12) *Spontaneous drawing:* the subject is required to draw on a blank sheet ‘*a daisy with the stem and a leaf’*. This test evaluates the ability to mentally retrieve and reproduce an image.

- 13) *Phonemic Fluency:* participant has to find as many words as possible starting with the same letter (C, P, S; 1 min each). This task requires cognitive flexibility in organizing and selecting lexical information, generation of a search strategy, and the ability to switch between categories inhibiting the exhausted one. This test evaluates executive functions.

- 14) *Cognitive Estimation*: it consists of five questions reflecting judgment and reasoning, such as: *How many camels live in Holland?* It assesses ability to make estimations and requires memory and executive functions, such as reasoning skills, problem solving, and judgment ability.

- 15) *Praxis ability:* it consists in a series of commands the subject has to execute using his/her hands and limbs. Two orders evaluate *ideomotor-apraxia*, the ability to carry out a motor command when using an object, i.e. a pantomime: *Act as if you were brushing your teeth.* Two other verbal orders evaluate *ideational apraxia*, the ability to create the idea of a specific movement, e.g., *show me the gesture to indicate “that man is crazy”*. Two items require the subject to copy a non-significant gesture shown by the examiner. In this case the subject has to plan and reproduce what he/she is observing.

- 16) *Clock drawing*: the subject is given a sheet of paper with a circle drawn on it, and s/he has to place all the numbers on the clock face and place the hands at 2:45. The test involves several cognitive abilities such as praxis, image retrieval, selection of numbers, planning number placement, and reasoning to place hands on the required time. Thus, executive functions are involved.

The *Activity of Daily Livin*g -ADL- scale [35] comprises the basic actions that involve caring for one’s self and body, including personal care, mobility, and eating. It is based on 6 criteria, each graded on the level of dependence (1 point if independent, 0 points if dependent).

The *Instrumental Activities of Daily Living -IADL- scale* [36] includes more complex skills related to independent living in the community (e.g., managing finances and medications, travelling independently, etc.). The self-report instrument (comprising 8 domains for women and 5 for men) with a summary score ranging from 0 (low function, dependent) to 8–5 (high function, independent), is most useful for characterizing how a person is functioning at the present time, and identifying improvement or deterioration over time. Assessment of level of independence in daily activities is paramount since the distinction between mild and major Neurocognitive Disorder (NCD) [39] is determined by the extent to which cognitive decline interferes with everyday functioning. In major NCD, or dementia, cognitive impairment influences independence in everyday functioning in a negative way. In mild NCD or MCI, individuals remain autonomous [40], although subtle problems may already occur in complex activities.

### Data analyses

All the items from the ENB, ADL, and IADL were initially submitted to the Rasch Model to evaluate whether different measures could be combined into a single global measure. Based on the items’ difficulty and subjects’ performance, the Rasch analysis provided the ability score (global functioning) for each subject. Participants’ ENB scores were obtained by summing the scores of the 16 tasks of the ENB. Each participant’s ADL, IADL, global functioning, and ENB scores, were then submitted to ANCOVAs, controlling for age and education, to test potential differences between groups with different levels of cognitive impairment. Based on the adjusted MMSE score, subjects were divided in three groups: (1) Severe Cognitive Impairment, MMSE adjusted score 0–17; (2) Moderate to Mild Cognitive Impairment, MMSE adjusted score 18–24; and (3) Normal Cognitive functioning, MMSE adjusted score > 24 [38].

Linear regressions were performed to evaluate the ability of ENB, ADL, IADL, and global functioning to predict cognitive impairment (MMSE score as dependent variable).

Finally, in order to test the ability of the instrument to classify subjects between groups with different cognitive impairment, we performed ROC (Receiver Operating Characteristic) curve analyses to compare the Areas Under the Curve (AUC) of global functioning, ENB, ADL, and IADL scores.

## Results

### Rasch analysis

As one of the limitations of the Rasch model is that it can only include ordinal or binary data, we transformed the score of the items that provided a continuous score into categorical data. The potential range of scores for each item was divided into four equal length categories. The possible categories for each item ranged between 1 (lowest performance; 0 to 25% of the maximum score) and 4 (highest performance; 75 to 100% of the maximum score). A total of 70 items (Table 1) were submitted to the Rasch model.

**Table 1:** List of the 70 items submitted into the Rasch model

Task	Score	Item Label
*Digit Span-DS*	0–1	ENB_DS_1–3 digits
	0–1	ENB_DS_2–3 digits
	0–1	ENB_DS_3–4 digits
	0–1	ENB_DS_4–4 digits
	0–1	ENB_DS_5–5 digits
	0–1	ENB_DS_6–5 digits
	0–1	ENB_DS_7–6 digits
	0–1	ENB_DS_8–6 digits
	0–1	ENB_DS_9–7 digits
	^a^0–1	ENB_DS_10–7 digits
	0–1	ENB_DS_11–8 digits
	^a^0–1	ENB_DS_12–8 digits
*Trail Making Test-A - TMT-A*	1–4	ENB_TMT_A
*Trail Making Test-B - TMT-B*	1–4	ENB_TMT_B
*Copy drawing - CD*	0–2	ENB_CD Copying a drawing of a house
*Interference memory test - IM*	1–4	ENB_IM_LP_1 Letters and positions of the sequence of letters (i.e. FGL) remembered after a mathematical calculation
	1–4	ENB_ IM _L_1 Letters of the sequence of letters (FGL) remembered after a mathematical calculation (regardless positions)
	1–4	ENB_ IM _LP_2 Letters and positions of the sequence of letters (PMT) remembered after a mathematical calculation
	1–4	ENB_ IM _L_2 Letters of the sequence of letters (PMT) remembered after a mathematical calculation (regardless positions)
	1–4	ENB_ IM _LP_3 Letters and positions of the sequence of letters (CRB) remembered after a mathematical calculation
	1–4	ENB_ IM _L_3 Letters of the sequence of letters (CRB) remembered after a mathematical calculation (regardless positions)
	1–4	ENB_ IM _LP_4 Letters and positions of the sequence of letters (ZLR) remembered after a mathematical calculation
	1–4	ENB_ IM _L_4 Letters of the sequence of letters (ZLR) remembered after a mathematical calculation (regardless positions)
	1–4	ENB_ IM _LP_5 Letters and positions of the sequence of letters (QVS) remembered after a mathematical calculation
	1–4	ENB_ IM _L_5 Letters of the sequence of letters (QVS) remembered after a mathematical calculation (regardless positions)
	1–4	ENB_ IM _LP_6 Letters and positions of the sequence of letters (DNC) remembered after a mathematical calculation
	1–4	ENB_ IM _L_6 Letters of the sequence of letters (DNC) remembered after a mathematical calculation (regardless positions)
*Abstract verbal reasoning - AVR*	0–2	ENB_AVR_1 Arm and leg are two …
	0–2	ENB_AVR_2 Laughing and crying are two …
	0–2	ENB_AVR_3 Eating and sleeping are two …
*Token test - TT*	0–1	ENB_TT_1 Touch a green token
	0–1	ENB_TT_2 Touch a yellow square
	0–1	ENB_TT_3 Touch the white square and then the green circle
	0–1	ENB_TT_4 Touch the white circle and then the red circle
	^a^0–1	ENB_TT_5 put the red circle on top of the green square
*Immediate recall prose memory - IMM*	1–4	ENB_MEMP_IMM Recalling information from a short story
*Delayed recall prose memory - DRPM*	1–4	ENB_DRPM Recalling information from a short story after overlapping figure test
*Overlapping figures - OF*	1–4	ENB_OF Distinguishing overlapping figures
*Spontaneous Drawing - SD*	0–2	ENB_SD Drawing a flower with a stem and a leaf
*Word phonemic fluency test - WPF*	1–4	ENB_WPF_C letter All the words that start with the letter “C”
	1–4	ENB_WPF_P letter All the words that start with the letter “P”
	1–4	ENB_WPF_S letter All the words that start with the letter “S”
*Cognitive estimation* – CE	0–1	ENB_CE_1 How much does a liter of fresh milk cost?
	0–1	ENB_CE_2 How far is Milan from Rome?
	0–1	ENB_CE_3 How long is a guitar?
	0–1	ENB_CE_4 How long is a mass?
	0–1	ENB_CE_5 How many kangaroos are there in the Netherlands?
*Ideative and ideomotor praxis test -IIP*	0–1	ENB_IIP-1 Pantomime the use of a hammer
	0–1	ENB_IIP-2 Pantomime the use of a toothbrush
	0–1	ENB_IIP-3 Gesture the meaning of a verbal instruction (the sign of the cross)
	0–1	ENB_IIP-4 Gesture the meaning of a verbal instruction (he/she is crazy)
	0–1	ENB_IIP-5 Copying meaningless gestures (middle finger arched on the index)
	0–1	ENB_IIP-6 Copying meaningless gestures (a forward arm with the palm of the hand open out, the other arm folded with a fist on the shoulder)
*Clock drawing test*	1–4	ENB_CDT_N (Numbers)
	1–4	ENB_CDT_D (Disposition/arrangement)
	1–4	ENB_CDT _HC (Hands of the clock)
*Activities of daily living - ADL*	0–1	ADL_1 – Bathing
	0–1	ADL_2 – Dressing
	0–1	ADL_3 - Toilet hygiene
	0–1	ADL_4 - Functional mobility
	0–1	ADL_5 – Continence
	^a^0–1	ADL_6 – Feeding
*Instrumental activities of daily living - IADL*	0–1	IADL_1 - Ability to use telephone
	0–1	IADL_2 – Shopping
	0–1	IADL_3 - Food preparation
	0–1	IADL_4 – Housekeeping
	0–1	IADL_5 – Laundry
	0–1	IADL_6 - Mode of transportation
	0–1	IADL_7 - Responsibility of own medications
	0–1	IADL_8 - Ability to handle finances

^a^Items removed because of a mean square higher than the accepted value of two. The final model consists of 66 items

The analysis of the fit statistics for the Rasch model, including the 70 (Table 1) items, showed that four items out of 70 (Digit Span trial 10, Digit Span trial 12, Token Test trial 5, and ADL item 5) did not fit the expectation of the model. Specifically, the four items showed, for the outlier-sensitive statistic (outfit) and for the information-weighted fit statistic (infit), a mean square higher than the accepted value of two. For this reason, four items were removed from the model, resulting in a final instrument of 66 items (Table 1).

Data were analyzed with the Winsteps software version 3.81.0. This software has a wide range of tools for the evaluation of data fit to the model, and provides estimates of person ability and item difficulty along with a common measurement continuum expressed in log-odd units (logits).

The analysis of the fit statistics for the Rasch model, including the 66 items (Table 1), showed that the resulting composite instrument was able to confirm the items’ difficulty hierarchy (Item Separation = 8.28), and to distinguish between patients’ ability levels (Person Separation = 2.67). Only 1% of the item measure (reliability 0.99) and 12% of the person measure variability (reliability 0.88) could be attributed to measurement error. The analysis of the dimensionality showed that the instrument explained 54.2% of the variance.

**Fig. 1:**
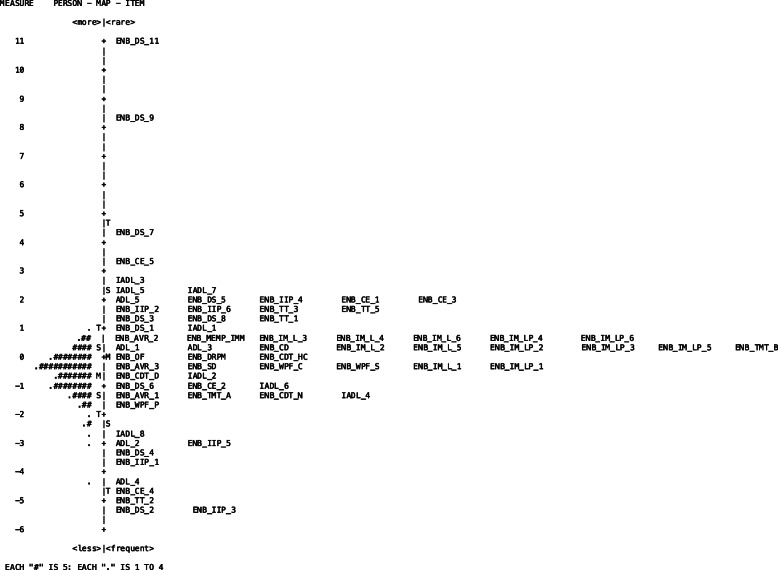
The person-item map shows how diverse measures of cognitive ability can be combined into a single measurement scale. If a person is shown at the same line as an item on the map, then that person is likely to pass that task half of the time. Two items on the same line, have the same level of difficulty

The items map (Fig. 1) shows how the study’s participants are located around 0, with subjects with lower ability located below 0, and subjects with normal-higher ability located above 0. Regarding the items, it is possible to notice that they tend to cluster in the lower part of the scale, suggesting that the items included in the model are more sensitive in discriminating subjects with lower ability. Only the digit span item 11 (8 digits) seems to require a noticeable ability compared to the others.

**Fig. 2:**
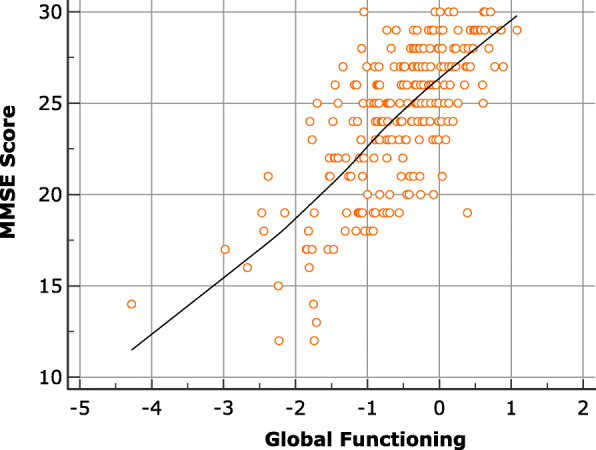
Correlation between MMSE and Global functioning

The ability score, or global functioning score, obtained with the Rasch analysis showed a positive correlation with the MMSE Score *r* = 0.715, *p* < .001 (Fig. 2).

### ANCOVAs results

Based on the adjusted MMSE score, subjects were divided in three groups: (1) Severe Cognitive Impairment, MMSE adjusted score 0–17; (2) Moderate to Mild Cognitive Impairment, MMSE adjusted score 18–24; and (3) Normal Cognitive functioning, MMSE adjusted score > 24 [38]. The global functioning score (ability score) of each subject, obtained through the Rasch analysis, ENB (overall score obtained by summing the 16 tasks of the ENB), ADL, and IADL scores were submitted to ANCOVAs – with age and education included as covariates in the analysis – to test potential differences between groups (Severe Cognitive Impairment vs Moderate to Mild Cognitive Impairment vs Normal Cognitive functioning) (Table 2).

**Table 2:** Group comparisons for Global Functioning, ENB, ADL and IADL

		Mean	SD	Estimated Marginal Mean	SE	95% CI		F	***p***	***ηp***^**2**^
						Lower Bound	Upper Bound			
**Global Functioning**	**Normal**	−0.20	0.56	−0.26	0.05	−0.35	−0.16	88.423	<.001	.407
	**Mild**	−0.88	0.63	−0.82	0.06	−0.93	−0.70			
	**Severe**	−2.11	0.73	−2.07	0.14	−2.34	−1.80			
**ENB**	**Normal**	45.51	20.11	43.74	1.37	41.05	46.44	37.24	<.001	.224
	**Mild**	26.98	14.19	29.26	1.62	26.07	32.44			
	**Severe**	14.35	8.34	15.43	3.91	7.74	23.13			
**ADL**	**Normal**	5.42	0.98	5.39	0.10	5.19	5.59	1.464	.233	.012
	**Mild**	5.23	1.19	5.27	0.12	5.04	5.50			
	**Severe**	4.88	1.76	4.90	0.28	4.36	5.45			
**IADL**	**Normal**	5.23	2.24	5.16	0.20	4.77	5.54	0.647	.525	.005
	**Mild**	4.71	2.23	4.83	0.23	4.37	5.29			
	**Severe**	4.79	2.52	4.75	0.59	3.58	5.92			

A main effect of the group was found for global functioning score, F_(2,263)_ = 88.423, *p* < 0.001, η2 = .407. Differences were then found between subjects with severe (M = − 2.07, SE = .14) and mild (M = −.81, SE = .06, *p* < .001) cognitive impairment; between subjects with mild cognitive impairment (M = −.81, SE = .06) and normal cognitive functioning (M = −.25, SE = .49, *p* < .001); and between subjects with normal cognitive functioning (M = −.25, SE = .49) and severe cognitive impairment (M = − 2.07, SE = .14, *p* < .001). A main effect of the group was also found for the ENB score, F_(2,263)_ = 37.240, *p* < 0.001, η2 = .224. Differences were found between subjects with severe (M = 15.43, SE = 3.91) and mild (M = 29.26, SE = 1.62, *p* < .005) cognitive impairment; between subjects with mild cognitive impairment (M = 29.26, SE = 1.62) and normal cognitive functioning (M = 43.74, SE = 1.37, *p* < .001); and between subjects with normal cognitive functioning (M = 43.74, SE = 1.37) and severe cognitive impairment (M = 15.43, SE = 3.91, *p* < .001). No main effect of the group was found for ADL, F_(2,263)_ = 1.464, *p* = .233, η2 = .012, and IADL, F_(2,263)_ = .647, *p* = .525, η2 = .005.

### Regressions

A linear regression, always controlling for age and education, showed that the global functioning score is a significant predictor of the MMSE Score, R^2^ = .512, β = .723, *p* < .001. Age (β = .017, *p* < .715) and Education (β = −.011, *p* < .800) were no significant predictors.

We then performed a linear regression using the classical ENB, ADL, and IADL total scores as predictors and the MMSE score as dependent variable. The resulting model was significant, R^2^ = .207, *p* < .001, with ENB (β = .597, *p* < .001) as significant predictor, while IADL (β = .026, *p* < .667), ADL (β = .001, *p* = .986), Age (β = .064, *p* < .287), and Education (β = .016, *p* < .777) were not significant predictors.

Although both regression models are the combination of the same items, when comparing the two regressions slopes, the composite score obtained by the Rasch model was a better predictor (*t* = 10.42, *p* < 0.001) of the MMSE score than the classical ENB, ADL, and IADL total scores.

This difference highlights the importance of considering the item’s difficulty that is taken into account only in the composite score obtained with the Rasch model.

### Receiver operating characteristic (ROC) curves: MMSE groups

In order to test the instrument ability to correctly re-allocate subjects to their diagnostic groups, we performed a ROC (Receiver Operating Characteristic) curve analysis to compare the Areas Under the Curve (AUC) of global functioning score, ENB, ADL, and IADL scores.

Both Global Functioning score (AUC = .949) and ENB score (AUC = .812) showed a significant AUC (*p* < .001) in the classification between severe and mild cognitive impaired subjects, with a greater AUC for global functioning score compared to the AUC for the ENB (*z* = 2.573, *p* < .01). ADL (AUC = .559) and IADL (AUC = .518) were not significant (*p* > .05). Sensitivity and specificity are reported in Table 3.

**Table 3:** Criterion values, sensitivity, and specificity for the ROC curves

		AUC	***p***	Criterion	Sensitivity	Specificity
**Mild-Normal**	**Global Functioning**	0.792	< 0.001	≤ −0.38	78.95	70.23
	**ENB**	0.778	< 0.001	≤34	76.84	70.99
	**ADL**	0.512	0.728	≤4	15.79	90.08
	**IADL**	0.572	0.058	≤4	48.42	65.65
**Mild-Severe**	**Global Functioning**	0.949	< 0.001	≤ − 1.15	100.00	87.37
	**ENB**	0.812	< 0.001	≤23	92.86	57.89
	**ADL**	0.559	0.447	≤2	14.29	97.89
	**IADL**	0.518	0.843	≤2	50.00	64.21

**Fig. 3:**
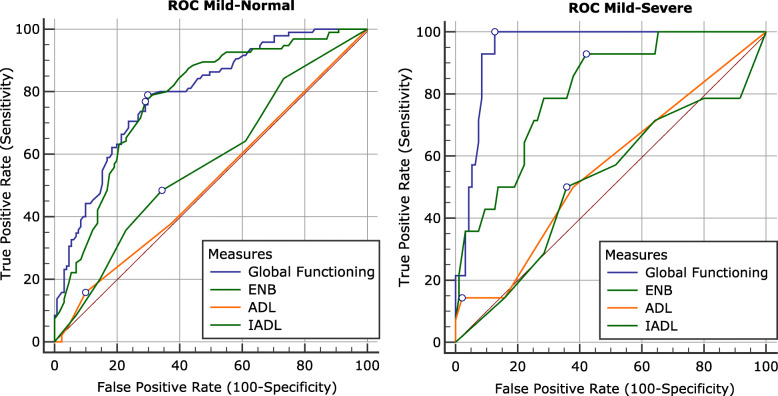
ROCs curves: MMSE Groups. Mild Cognitive Impairment vs Normal Cognitive functioning and Severe Cognitive Impairment vs Mild Cognitive Impairment. Criterion value is indicated on each curve

In the classification between mild cognitive impaired subjects and subjects with normal cognitive functioning (Fig. 3), both global functioning score (AUC = .792) and ENB (AUC = .778) showed a significant AUC (*p* < .001). No significant difference between global functioning score and ENB was found (*z* = .571, *p* = .568). ADL (AUC = .512) and IADL (AUC = .572) were not significant (*p* > .05). Sensitivity and specificity are reported in Table 3.

### Global functioning score and clinical groups

In order to test the ability of the global functioning score to classify the individuals based on their diagnosis, we divided our sample in: 1) Individuals with normal cognitive functioning; 2) Mild Neurocognitive Disorder; and 3) Major Neurocognitive Disorder [40]. The Mild Neurocognitive Disorder group included individuals with impairment in one or more cognitive domains who preserved independence in functional abilities. The Major Neurocognitive Disorder group included individuals with impairment in one or more cognitive domains with compromised independence in functional abilities [40, 41].

**Fig. 4:**
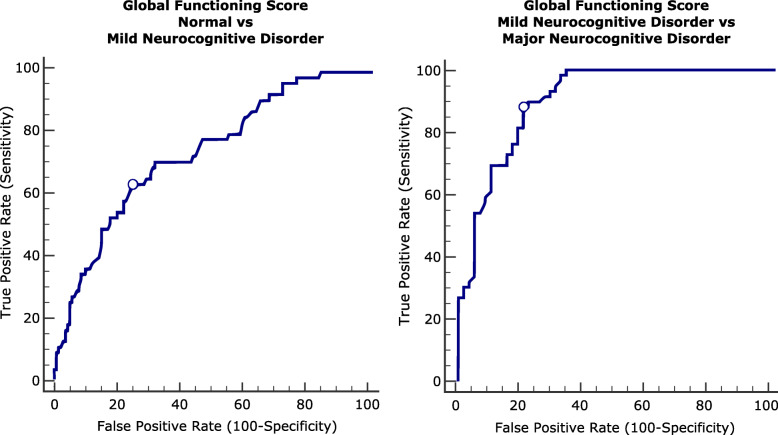
ROCs curve: Clinical Groups. Mild Neurocognitive Disorder vs Normal Cognitive functioning and Major Neurocognitive Disorder vs Mild Neurocognitive Disorder. Criterion value is indicated on the curve

In the classification between subjects with Mild Neurocognitive Disorder and subjects with normal cognitive functioning (Fig. 4), the global functioning score (AUC = .751) showed a significant AUC (*p* < .001) with a sensitivity of 66.10 and a specificity of 74.83 (criterion <= − .52). In the classification between subjects with Mild Neurocognitive Disorder and subjects with Major Neurocognitive Disorder (Fig. 4), the global functioning score (AUC = .905) showed a significant AUC (*p* < .001) with a sensitivity of 88.14 and a specificity of 79.66 (criterion <= − 1.26).

## Discussion

With the rise of the dementia global health crisis, utilization of evidence-based outcome measures to determine optimal care is crucial. Diagnosis of incident dementia, or assessment of cognitive impairment severity, is essential to evaluate potential intervention effects. Therefore, standardization and implementation of appropriate outcome measures, for patients with cognitive impairment, will decrease the considerable variability in the evaluation of this population. However, it is still undetermined as to which cognitive test, or tests, provide the best power and should be used as the primary efficacy endpoint in intervention trials. Recent secondary preventive randomized controlled trials (like the Alzheimer’s Prevention Initiative) [42], that enroll asymptomatic individuals with preclinical AD and early symptomatic individuals, propose to employ different cognitive endpoints for their efficacy analysis, typically in the form of composite cognitive score, with equal weights over several cognitive tests [43].

In this study we proposed the development and evaluation of a new approach, using the Rasch model, for measuring global functioning (that is the overall ability of patients to function in their everyday activities) in older adults with cognitive impairment and dementia.

Optimal measures should, in fact, demonstrate bi-directional sensitivity and be able to detect long-term stability, as a successful outcome of preventive interventions, and worsening in case of treatment failure. Sensitivity to change is also crucial for longitudinal tracking and sensitivity to impairment, which is essential to detect signs of incipient illness [3]. Further, the identification of a single score has, in fact, a series of advantages such as being easily comparable at different time points and across the range of cognitive impairment severity levels. Here, we combined different neuropsychological batteries and cognitive-related tasks from the ENB battery, ADL, and IADL questionnaires, commonly used in clinical practice to diagnose cognitive impairment in older adults in Italy, into one single scale using Rasch analysis. The resulting measure provided by the Rasch analysis showed that all the considered measures are part of one single dimension indexing one single construct (i.e. global functioning) and that different tasks could be combined into one single linear dimension, i.e. cognitive ability, explaining 54.2% of the variance.

The instrument (global functioning score) derived from these tests showed a good item and person separation, indicating that the selected items are able to discriminate the subjects’ ability. From the item map (Fig. 1) it is possible to notice that the − 66- items included in our analysis tend to cluster in the lower part of the scale, suggesting higher sensitivity in discriminating between subjects of lower ability. It is important to remark that, items in the lower part of the scale are considered “easier” compared to items located in the higher part of the scale, therefore requiring less ability. This result suggests that more difficult tasks are needed in order to better discriminate among subjects with higher ability in pre-symptomatic or prodromal stages of the disease. Such a pattern of findings is very informative, as it implies that classic cognitive/functional tests and scales might not show the expected sensitivity to impairment, which is necessary to discriminate incipient illness from lifelong premorbid limitations in functioning. However, dimensionality analysis indicates that the included − 66- items, comprising cognitive and functional measures, can be considered as part of one single dimension (unidimensional), indexing the same hidden trait (i.e. impairment). Again, this finding bears important clinical implications since it demonstrates that performance-based identification of impairment should include assessments of both cognition and functional capacity.

Moreover, as the model was able to map each item’s raw score into a level of difficulty scale, segregating them to the level to which they belonged, it identified key elements for the evaluative procedure. For example, the items-map of our model showed that several items of the ENB, ADL, and IADL belonged to the same difficulty level (same line on the map), suggesting the possibility of reducing the number of items administered during the assessment phase with evident advantages for clinicians.

Therefore, the use of such a global functioning score can solve a critical point in clinical practice related to different and contradictory outcomes, or patient “identification” / categorization/profile, that result from the different tests. In our case, ADL and IADL, in contrast to the ENB, did not show a significant difference between the groups (severe, mild, and normal individuals). The global functioning score, obtained through the Rasch model, instead, provided an easier interpretation of the changes in the overall ability of the subject due to both- the ability to combine cognitive and functional measures in one single score, and the ability of the instrument to consider the difficulty of the items, thus solving the above mentioned contradiction and allowing group difference to emerge.

The ROC results also showed a better classification performance for the combined measure (global functioning) compared to the ENB alone, particularly for discriminating between subjects with severe and mild cognitive impairment. These results seem to be explained by the items included in the model. In fact, the item map provided by the Rasch model showed that our items were located below 0, suggesting a better discrimination ability of subjects with lower ability. Based on the data from the ROC curves, results show that in the classification Normal vs Mild, the global functioning score did not differ, in terms of sensitivity and specificity, from the ENB (a cognitive measure). This result seems to be in line with the literature suggesting that in an early stage of the disease only the cognitive component is affected, and therefore, a cognitive measure alone can efficiently identify patients [41]. Conversely, results completely change when the same measures are used to discriminate Mild from Severe patients. As we know from the DSM 5 criteria [40], Major neurocognitive disorder is characterized by a decline in mental ability severe enough to interfere with independence and daily life. It is thus crucial, in the clinical practice, to take into account both cognitive and functional measures in order to diagnose a major neurocognitive disorder. Our results seem to support the international diagnostic criteria. The ROC curves show that the global functioning score (a combined cognitive and functional measure) has a better sensitivity and specificity, for the classification of Mild vs Severe groups, compared to the ENB (a cognitive measure), ADL, and IADL (both functional measures) considered separately. These results suggest that, in an advanced stage of the disease, cognitive measures alone, or functional measures only, are not sufficient for the diagnosis. A combined evaluation, of both cognitive and functional domains is needed. The global functioning score offers the possibility of looking at both domains at the same time and might constitute a good endpoint in clinical trials.

All together, these results suggest that the diverse cognitive-related measures can be considered part of one single dimension (global functioning) and, furthermore, that this dimension can be measured as a single construct and score. Future studies should test the ability of the global functioning score to monitor the progression of the disease, or potential changes after an intervention, in order to establish the “long-term” sensitivity and specificity of the instrument. This study was based on retrospective data, and therefore, follow-up data to monitor progression were not available. Nonetheless, the global functioning score can be easily generated for each subject at different timepoints if data are available. Future studies should also include people with functional impairment due to physical inability, not considered here, to assess the “relationship” between physical impairment and functional impairment.

## Conclusions

Although previous studies have proposed composite measures (cognitive and functional) as endpoints for longitudinal cognitive-functional changes in elderly population [44, 45], these measures are often based on independent evaluation of behavioral observations of both functional and cognitive domains. This study provides empirical evidence of the benefits derived from the implementation of a score that takes into account a more comprehensive evaluation of the elderly patients’ overall cognitive and functional ability based on the subjects’ performance and the items’ level of difficulty. The global functioning score here proposed offers, at least, two major novelty aspects:

1. the score is determined by the direct performance of the individual for the cognitive and functional tasks;
2. each item in the global functioning score is weighted based on its difficulty. For example, our data showed that food preparation is more cognitive demanding than using the phone.

While this study does not aim to provide a final instrument for immediate use, it offers an alternative perspective for future development of instruments that would help clinicians in measuring global functioning in patients at different stages of cognitive impairments and different baseline level of performance. This approach, using Rasch model, could provide future guidelines for considering the personal ability in respect to the level of difficulty of a particular item when the impact of cognitive impairment on elderly patient’s global functioning needs to be determined.

## Data Availability

The datasets analyzed during the current study available from the corresponding author on reasonable request.

## Abbreviations

MMSE: Mini Mental State Examination
ENB: Esame Neuropsicologico Breve
ADL: Activities of Daily Living
IADL: Instrumental Activities of Daily Living
ROC: Receiver Operating Characteristic
MCI: Mild Cognitive Impairment
M: Mean
SD: Standard Deviation
SE: Standard Error
TMT A: Trail Making Test A
TMT B: Trail Making Test B
AUC: Areas Under the Curve
ANCOVA: Analysis of covariance
DSM: Diagnostic and Statistical Manual of mental disorders
NCD: Neurocognitive Disorder
